# Case Report: A case series on actionable genomic alterations guiding effective targeted therapy in rare sarcomas

**DOI:** 10.3389/fonc.2026.1842108

**Published:** 2026-06-29

**Authors:** Jamie L Zeal, Mark G Woodcock, Kevin Y Chen, Jacob N Stein

**Affiliations:** 1 Division of Pediatric Hematology-Oncology, Department of Pediatrics, University of North Carolina, Chapel Hill, NC, United States; 2 Division of Oncology, Department of Medicine, University of North Carolina, Chapel Hill, NC, United States; 3 Lineberger Comprehensive Cancer Center, University of North Carolina, Chapel Hill, NC, United States; 4 University of North Carolina Medical Center, Department of Pharmacy, Chapel Hill, NC, United States

**Keywords:** ALK, case report, homologous recombination deficiency (HRD), next generation sequencing (NGS), precision oncology, ROS1, sarcoma, targeted therapy

## Abstract

**Background:**

Advanced sarcomas have limited systemic therapy options, but genomic profiling can uncover actionable mutations. This case series illustrates the promise and limitations of next-generation sequencing (NGS) and targeted therapies in clinical practice across three sarcoma histologies.

**Case presentations:**

Case 1: A 36-year-old woman received local therapies for an angiosarcoma in her breast. Six months later, she developed osseous metastatic disease, and NGS revealed a *GOPC-ROS1* fusion. She had a partial response to crizotinib. This ROS1 inhibitor and one course of radiation for oligometastatic progression controlled her disease for 12 months. Subsequent lines of therapy included pembrolizumab for high PD-L1 status, with suspected hyperprogression. Repotrectinib was trialed to overcome her *ROS1 G2032R* resistance mutation, but it had limited efficacy. She ultimately succumbed to her disease. Case 2: A 30-year-old woman with Neurofibromatosis Type 1 developed a high-grade, ALK-rearranged peripheral malignant nerve sheath tumor at her left antecubital fossa. Following resection, pulmonary metastases were identified. RNA transcriptome analysis revealed an *EML4-ALK* rearrangement. She had a prolonged partial response to alectinib. The ALK inhibitor and two courses of radiation for oligoprogression have continued to control her disease at 48 months. Case 3: A 51-year-old woman with uterine leiomyosarcoma developed pulmonary metastases shortly after completing adjuvant radiation. NGS revealed a BRCA2 deep deletion. After progressing on multiple lines of systemic therapies, her widely metastatic disease has been controlled for 62 months with niraparib and local therapies for oligoprogression.

**Discussion:**

Three patients with advanced sarcomas achieved partial responses to NGS-guided targeted therapies, with disease control lasting from 1 to over 5 years. Two cases involved novel, targetable mutations not previously identified in these sarcoma subtypes. However, the limitations of targeted therapies are highlighted by one patient’s lack of response to a novel ROS1 G2032R-directed treatment, and another patient’s repeated oligometastatic progression despite using an ALK inhibitor without resistance mutations. Simply suppressing a driver-mutation clone with targeted therapy may be insufficient for long-term disease control in sarcoma. Local therapies can be critical adjuvants in achieving improved outcomes for aggressive sarcomas. We recommend careful consideration of NGS testing for all sarcomas as we continue to explore their vast mutational landscape, response to targeted therapies, and mechanisms of resistance.

## 1 Introduction

Soft tissue sarcoma (STS) ushered in the era of precision medicine to the solid oncology space. The seminal paper by Heinrich et al. (1) showed that *KIT*-mutant gastrointestinal stromal tumor (GIST) responded to a tyrosine kinase inhibitor (TKI), imatinib, a finding subsequently replicated across numerous cancer types and oncogenic pathways. In the decades that followed, the targeted approach to GIST has been further refined, with seven TKIs now approved for the treatment of the disease (2). There is a rapidly expanding armamentarium of targeted therapies for other malignancies, such as non-small cell lung cancer (NSCLC) and breast cancer. However, this success has not been replicated across the field of sarcoma. Several agents have been approved for individual, often rare histologies, such as the CSF1R inhibitors pexidartinib (3) and vimseltinib (4) for tenosynovial giant cell tumor; and tazemetostat for epithelioid sarcoma (5). The efficacy of palbociclib for differentiated liposarcoma is acknowledged by NCCN guidelines (6, 7). *NTRK* fusions are a promising target across sarcoma subtypes, and inhibition offers clear benefit compared with other treatment options (8). Yet, there remains a paucity of effective therapeutic options to treat advanced disease in most STS subtypes. Nearly half of patients with STS will develop metastatic disease, with a median overall survival of 12 months (9–11). Improved systemic therapies for these conditions are imperative.

The use of somatic next-generation sequencing (NGS) has the potential to refine precise histologic diagnoses in sarcoma and identify a range of targetable mutations (15, 16). However, a common challenge across cancer types is the translation of actionable mutations identified on NGS into clinical benefit (17). In a large study of 7,494 sarcoma samples across 44 histologies, NGS refined 10.5% of diagnoses and identified at least one potentially actionable mutation (per OncoKB) in 31.7% (2372) of cases. FDA approval for the identified agent use in that sarcoma subtype was limited to only 5.9% (439) (16). Treating physicians at Memorial Sloan Kettering Cancer Center considered their sarcoma patients’ genomic profiling results actionable in 47% cases, with 29% of the cohort enrolled in a matched trial or off-label use of an FDA-approved medication (16). The authors note several durable partial responses to NGS-informed therapies in patients with refractory sarcomas (16). Here, we present a case series of patients with STS treated with individualized targeted therapies based on their tumors’ specific molecular profiles. In doing so, we hope to illustrate both the promise and limitations of this approach.

## 2 Methods

Written consent was obtained from the selected patients or their living representative for the publication of their oncologic diagnosis and treatment details, including pertinent genomic testing, imaging, and attempts to access clinical trials. Written consent was obtained for off-label use of alectinib. This case series is considered Non-Human Subjects Research by the University of North Carolina’s Office of Human Research Ethics policies and therefore did not meet the criteria for Institutional Review Board oversight. Individual charts were retrospectively abstracted to capture relevant clinical data, including diagnosis, NGS results, treatments received, and outcomes. Radiographic responses were independently assessed by the treating provider using clinical judgment with additional review of radiologists’ interpretation for concurrence. Treating physicians ordered StrataNGS (18), Tempus xT (19), or Tempus xF (20) based on shifting institutional partnerships with these commercially available, validated somatic NGS panels. Guardant360 CDx (21, 22) was used per provider preference. Geneticists and genetic counselors guided the acquisition of germline hereditary cancer testing. Results of genomic testing are summarized in **Table 1**.

**Table 1:** Summary of genomic testing results.

Case	Platform	Sample characteristics		Genomic findings	Biomarker Findings		
		Source (location)	tumor content (%)	CNV, indels, and fusions reported	TMB (mut/Mb)	MSI	PD-L1 (RNA expression score)
1	Invitae Common Hereditary Cancers panel, Invitae Sarcoma, Preliminary-evidence Genes for Sarcoma	blood (germline)	NA	No pathologic mutations detected.	NA	NA	NA
	StrataNGS version 3; Bioinformatics pipelines version 3.8	tissue (breast)	55%	GOPC-ROS1 fusion	Low (2)	MSS	High (49)
	Guardant360 CDx	blood (ctDNA)		ROS1 G2032R mutation (VAF 0.2%)		ND	
2	Tempus xT 648 gene version 4; Pipeline version 3.2.1	tissue (arm)	50%	EML4-ALK rearrangement; Pathogenic: CDKN2A (copy number loss), CDKN2B (copy number loss), MUTYH (c.536A>G, p.Y179C, Missense LOF, VAF 41.4%), NF1 (c.3997G>T, p.E1333*, Stop gain LOF, VAF 67.8%), PRDM1 (p.R25fs, c.75del, frameshift LOF, VAF 67.5%)	Low (7.9)	MSS	NR
	Tempus xF 105 gene liquid biopsy; pipeline version 5.2.0	blood (ctDNA)		NF1 (c.3997G>T, p.E1333* Stop gain - LOF; VAF 46.9%)		ND	
	Tempus xT 648 gene version 4; Pipeline version 3.6.0	tissue (lung)	60%	EML4-ALK rearrangement; Pathogenic: ALK (copy number gain); CDKN2A (copy number loss), CDKN2B (copy number loss), MUTYH (c.536A>G, p.Y179C, Missense LOF, VAF 45.9%), NF1 (c.3997G>T, p.E1333*, Stop gain LOF, VAF 71.5%), PRDM1 (p.R25fs, c.75del, frameshift LOF, VAF 73.5%)	Low (8.9)	MSS	NR
	Invitae Hereditary Breast and Gyn Cancers Panel, Selected genes; MUTYH single gene testing	blood (germline)	NA	Pathogenic mutations: NF1 (heterozygous, c.3997G>T, p.Glu1333*); MUTYH (heterozygous, c.536A>G, p.Tyr179Cys)	NA	NA	NA
3	StrataNGS version 3; Bioinformatics pipelines version 3.8	tissue (vertebral body)	75%	ERBB2 p.R896H (VAF 51%); BRCA2 deep deletion (sub-gene deletion, exons 2-18); RB1 deep deletion (sub-gene deletion, exons 19-27); TP53 p.W91Sfs*56 (VAF 76%)	Low (1)	MSS	Low (6)

## 3 Case descriptions

### 3.1 Case 1: ROS1-mutated angiosarcoma

**Figure 1:**
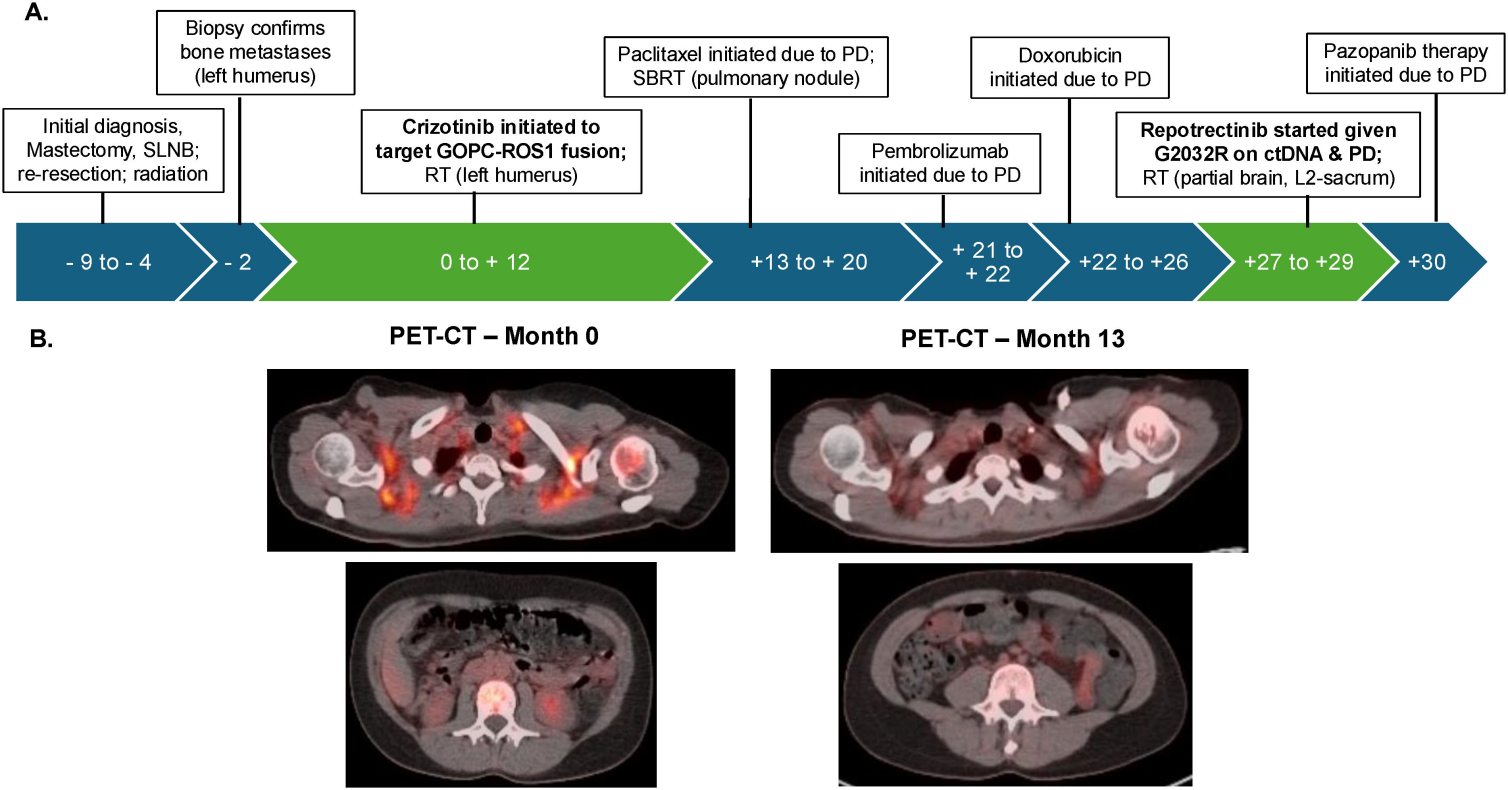
Case 1, primary angiosarcoma with *ROS1 G2032R* mutation, high PD-L1 status – 1A. Timeline of diagnosis and cancer-directed therapies. Time points are anchored to the initiation of crizotinib (Month 0). Pre-crizotinib clinical history is denoted in negative months, and post-initiation therapy is denoted in positive months. 1B. PET CT demonstrating partial response to crizotinib at three months. ctDNA, circulating tumor DNA; NA, not applicable; ND, not detected; NR, not reported; SLNB, sentinel lymph node biopsy; PET-CT, positron emission tomography with computed tomography; PD, progressive disease; RT, radiotherapy.

HD was a 36-year-old woman with an acute onset of right breast pain, with rapid development of erythema and swelling. No improvement with antibiotics. Ultrasound and mammogram showed large masses in the upper outer and retroareolar regions (**Figure 1**). Biopsies of both sites revealed primary angiosarcoma and associated hemorrhage. She underwent mastectomy (tumor measured 12.5 x 9.5 cm) and sentinel lymph node biopsy (0/3 nodes positive). Breast tissue demonstrated PD-L1 IHC >1% (2% immune cells stained, IC intensity 2+; PD-L1 SP142 FDA Tecentriq for TNBC (Breast)). Due to a potential positive margin despite re-excision, adjuvant radiation was added (IMRT, right chest wall 54 Gy/27 fractions; upper internal mammary, supraclavicular, and axillary nodes 48.6 Gy/27 fractions; right mastectomy scar total with boost 64 Gy/32 fractions). PET CT scan was unremarkable. Concurrent chemotherapy with paclitaxel was discussed, but it was not pursued.

Given her rare cancer diagnosis and her son’s NF1 diagnosis, she was screened for a cancer predisposition syndrome (Invitae Sarcoma panel and Common Hereditary Cancers Panel; total of 61 genes analyzed), but testing did not reveal pathologic germline mutations.

Two months later, two sites of osseous metastatic disease (L2, left humeral neck) were noted on PET CT scan and confirmed with a biopsy. NGS (StrataNGS, 55% tumor content from breast mass) revealed a *GOPC-ROS1* fusion on RNA transcriptome analysis; biomarker analysis showed microsatellite stability, low tumor mutational burden (2 mut/Mb), and high PD-L1 status (RNA expression score 49). Crizotinib and entrectinib were considered for targeting the *ROS1* fusion; the former, with less CNS penetration, was selected for its lower incidence of CNS side effects and rarity of CNS metastases in sarcoma. Restaging PET CT prior to initiation of targeted therapy revealed numerous bone metastases in the appendicular and axial skeletons (left scapula, left humeral head, lateral left second rib, posterior left fifth rib, right seventh rib, L2 vertebral body), so denosumab was added soon thereafter. She was initiated on crizotinib 250 mg twice daily as a first-line therapy. She had decreased FDG uptake on subsequent PET CT, with no new hypermetabolic lesions. Eight months after the initiation of crizotinib therapy, she developed oligometastatic progression with pain and growth of a left humeral head lesion. She underwent palliative radiation (left humeral head, 3D CRT, 20 Gy/5 fractions) and continued crizotinib until she had clear systemic progression five months later with numerous hypermetabolic lung nodules (up to 1.4 cm), paraesophageal mass (1.3 x 0.6 cm), and increased FDG uptake of bony lesions.

She then received further radiation to her humerus (distal left humerus, 3D CRT, 20 Gy/5 fractions), while paclitaxel controlled her disease for five months before she had oligoprogression of a pulmonary nodule. She received focal lung radiation (right lower lobe, IMRT, 60 Gy/8 fractions) and continued paclitaxel for three additional months until more widespread progression.

A clinical trial with nivolumab and cabozantinib (Alliance A091902, NCT04339738) was investigated, but it was closed to accrual. Pembrolizumab was recommended based on high PD-L1 status and responses observed in early-phase trials and case series, though most benefit was seen in cutaneous angiosarcomas of the head and neck and combination therapies (23–26). Restaging scans after a delay in securing the drug demonstrated increased disease burden, including enlarging left-sided pulmonary nodules with new right-sided pleural thickening/nodularity and large pleural effusion. Over the first six weeks of pembolizumab therapy, she developed worsening of her known large malignant pleural effusion that, despite serial thoracenteses (1500 mL removed x3), ultimately led to hospitalization with respiratory failure. Chest CT following emergent chest tube placement showed increased pleural disease burden (18 mm nodule, previously 9 mm) and effusion, causing complete right lung atelectasis and mediastinal shift, and growing lung nodules (34 mm x 30 mm, previously 20 mm x 16 mm). Despite the preventative effect of high PD-L1 status (27), hyperprogression from immunotherapy was suspected. There is ongoing work to address the challenge of distinguishing aggressive disease biology from hyperprogression, with most scoring systems relying on RECIST-based measurements for tumor growth acceleration calculations and some incorporating performance status and time from initiation of immunotherapy (28). Her CT imaging bookending an antecedent systemic therapy delay provides some insight into her rate of natural disease progression, but calculations are less precise with non-measurable malignant effusions and bony metastases. Also, her concurrent *Streptococcal* bacteremia could have contributed to her worsening clinical status. During hospitalization, she started doxorubicin while awaiting ROS1-inhibitor resistance testing and had clinical improvement and a partial disease response.

Tissue biopsy was unsuccessful; however, peripheral blood ctDNA sequencing (Guardant360 CDx) revealed a *ROS1* G2032R mutation (VAF 0.2%). Based on NGS results, a novel ROS1 inhibitor, repotrectinib, was pursued for compassionate use, given activity observed in early-phase data in non-small cell lung cancer with *ROS1 G2032R* resistance mutations (29). While awaiting drug approval, she developed brain metastases, which were treated with radiation (partial brain, 3D CRT, 30 Gy/10 fractions), steroids, and gemcitabine. She concurrently received palliative radiation for lower back pain (L2-sacrum, 3D CRT, 8 Gy/1 fraction). She initiated repotrectinib 160 mg twice daily, but she had progressive disease after 3 months on therapy. She was briefly trialed on pazopanib before transitioning to hospice care.

### 3.2 Case 2: ALK-rearranged malignant peripheral nerve sheath tumor

**Figure 2:**
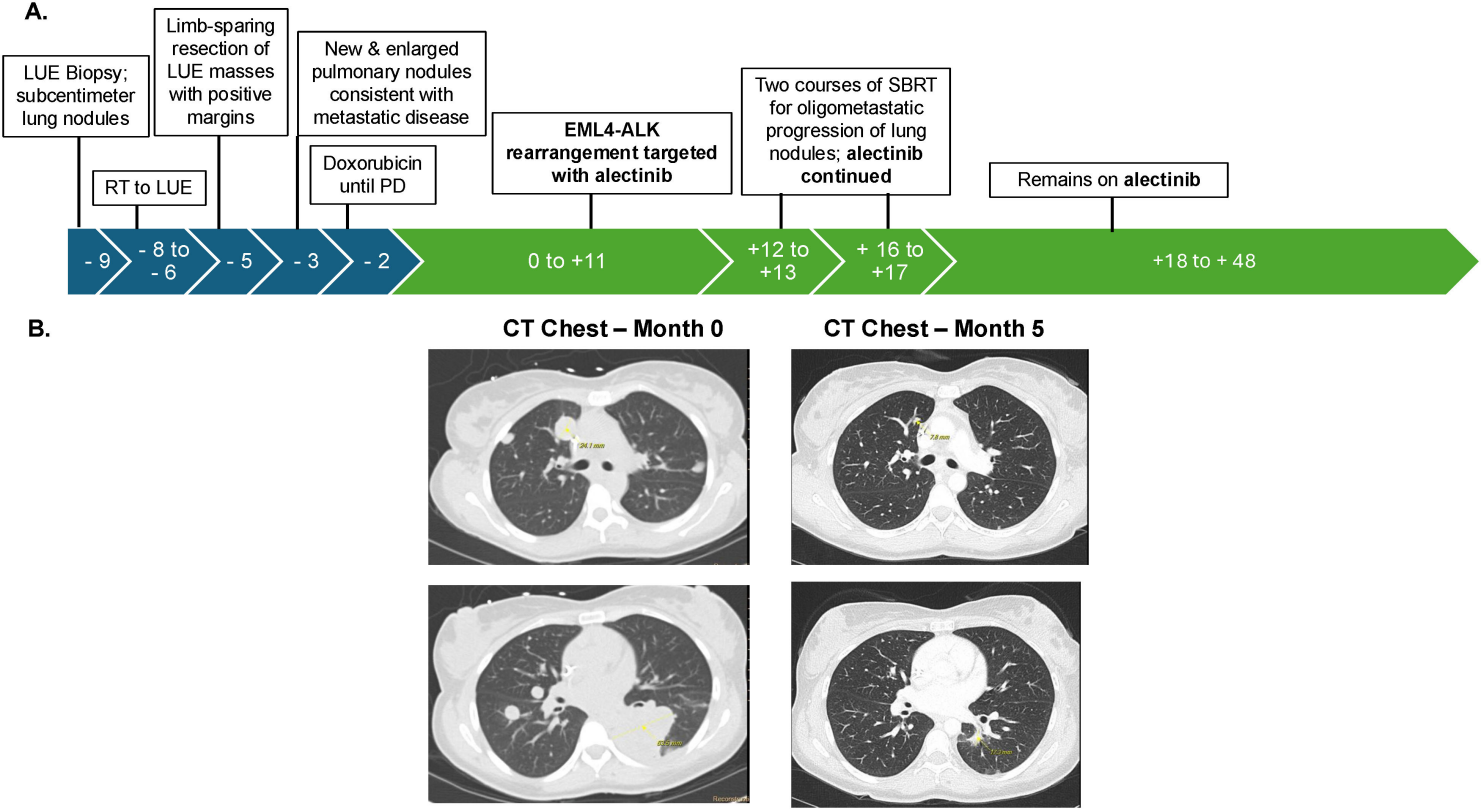
Case 2, NF1 with LUE malignant peripheral nerve sheath tumor and pulmonary metastases - **(A)** Timeline of diagnosis and cancer-directed therapies. Time points are anchored to the initiation of alectinib (Month 0). Pre-alectinib clinical history is denoted in negative months, and post-initiation therapy is denoted in positive months. Her disease remains well controlled on alectinib augmented by local RT at 48 months. **(B)** CT chest demonstrating partial response to alectinib sustained at five months. CT, computed tomography; LUE, left upper extremity; PD, progressive disease; RT, radiotherapy; SBRT, stereotactic body radiation therapy.

SR was a 30-year-old woman with neurofibromatosis type 1 (NF1), with sequelae limited to plexiform neurofibromas, when she sought evaluation for worsening neuropathy and pain along with hardening and enlargement of her left antecubital neurofibroma (**Figure 2**). A PET scan showed an antecubital mass and enlarged axillary lymph nodes; subsequent biopsy revealed high-grade MPNST. She underwent oncologic resection with negative margins and received radiation (left elbow, 3D CRT, 50 Gy/25 fractions).

Her first re-staging scan showed new and growing pulmonary nodules. She was treated with upfront doxorubicin chemotherapy while NGS (Tempus xT 648 gene panel, 50% tumor content from arm mass) was sent, which revealed an *EML4-ALK* rearrangement on RNA transcriptome analysis; biomarker analysis showed microsatellite stability and low tumor mutational burden (7.9 mut/Mb). Her disease progressed through doxorubicin. We attempted to enroll her on the national TAPUR (NCT02693535) Phase II clinical trial with crizotinib, but there was no trial availability for her specific mutation.

Thus, the patient consented to our recommendation for off-label alectinib 600 mg twice daily based on efficacy seen in ALK-rearranged NSCLC (30) and NCCN guidelines for ALK-rearranged inflammatory myofibroblastic tumors (31). During the three months it took to secure an affordable medication supply, she developed symptomatic progression of her pulmonary disease. After starting alectinib, she had a partial response, with over 50% decrease in size of many pulmonary nodules (largest was 6.4 cm, now 2.0 cm) and others completely resolved at six weeks. Eight months later, she had oligometastatic progression of an isolated pulmonary nodule; she remained on alectinib and was treated with targeted radiation (right lung, Linac-based SBRT, 50 Gy/5 fractions). Three months later, she again had oligometastatic progression of a different pulmonary nodule with mildly symptomatic pulmonary obstruction.

A fine needle aspirate was obtained for resequencing (Tempus xT 648 gene panel, 60% tumor content from lung), which again revealed an *EML4-ALK* rearrangement on RNA transcriptome analysis, but no new resistance mutations; biomarker analysis showed microsatellite stability and low tumor mutational burden (8.9 mut/Mb). She again received targeted radiation (left perihilar mass, IMRT, 45 Gy/15 fractions) and retains her prolonged disease control as of her last clinical evaluation, 48 months from initiation of alectinib.

SR’s maternal grandmother had no clinical findings associated with NF1 and developed breast cancer in her 40s. SR’s germline testing was consistent with her clinical NF1 diagnosis and tumor sequencing. Results showed pathogenic mutations in NF1 (heterozygous, c.3997G>T, p.Glu1333*) (Invitate Hereditary Breast and Gyn Cancers Panel (22 genes) and MUTYH (heterozygous, c.536A>G, p.Tyr179Cys) (Invitae single gene testing).

### 3.3 Case 3: BRCA-deficient leiomyosarcoma

DD was a 51-year-old woman with abnormal uterine bleeding managed with leuprolide, when she underwent evaluation of hypercalcemia and elevated parathyroid-related hormone. Final pathology from her total abdominal hysterectomy confirmed a large, locally advanced (stage II) leiomyosarcoma of the uterus and parametrial nodule (**Table 2**). She was treated with adjuvant gemcitabine and docetaxel.

**Table 2:** **Case 3: uterine leiomyosarcoma with ERBB2 mutation, BRCA2 deep deletion, RB1 deep deletion, TP53 mutation** – Timeline of cancer-directed local and systemic therapies.

Months relative to niraparib initiation	Site	Therapeutic intervention
-69	uterus	Total abdominal hysterectomy & bilateral salpinoophorectomy
-66 to -68	NA	gemcitabine & docetaxel
-54 to -57	NA	doxorubicin
-46	RUL pulm nodule	CT-guided RFA
-43	RUL pulm nodule	CT-guided RFA
-43	3 LUL pulm nodules	Cyberknife, 30 Gy/5 fractions
-39 to -35	NA	pazopanib on ALLIANCE A091304
-35 to -33	NA	sapanisertib on ALLIANCE A091304
-29	LUL paraspinal lesion	SBRT/Cyberknife, 30 Gy/5 fractions
-29	RLL pulm nodule	SBRT/Linac, 50 Gy/5 fractions
-26	T12-L3	3D-CRT, 30 Gy/10 fractions
-19	L1	Pathologic fracture repair: Partial L1 corpectomy, posterior T11-L3 instrumented fusion, open reduction of L1 vertebral body fracture, cement augmentation of L1 vertebral body, and laminectomy for excision of extradural neoplasm
-19	L1	SBRT/Cyberknife, 30 Gy/5 fractions
-18	perisplenic, anterior abdominal, SC nodules	CT-guided cryoablation with hydrodissection
-15	left second rib	3D CRT, 30 Gy/10 fractions
-9	C2	Pathologic fracture with spinal cord compression repair: C1-4 posterior fusion, C1-3 laminectomy with resection of intraspinal epidural and prevertebral tumor at C2
-8	six brain lesions	SRS/CyberKnife, 18-20 Gy/1 fraction
-8	C2	SBRT/Cyberknife, 25 Gy/5 fractions
-4	four SC nodules	CT-guided cryoablation with hydrodissection
-3	NA	temozolamide
-2	NA	trabectedin
-2	left femoral neck	3D-CRT, 0.8 Gy/1 fraction
0	NA	niraparib
1	left femoral neck	3D-CRT, 20 Gy/5 fractions
8	four SC nodules	CT-guided cryoablation with hydrodissection
13	four SC nodules	CT-guided cryoablation with hydrodissection

3D-CRT, three-dimensional conformal radiation therapy; CT, computed tomography; LUL, left upper lobe; PD, progressive disease; NA, not applicable; pulm, pulmonary; RUL, right upper lobe; RT, radiotherapy; RFA, radiofrequency ablation; SBRT, stereotactic body radiation therapy; SC, subcutaneous; TAH/BSO, total abdominal hysterectomy and bilateral salpingo-oophorectomy; VIR, vascular and interventional radiology.

Time points are anchored to the initiation of niraparib (Month 0). Pre-niraparib therapies are denoted in negative months, and post-initiation therapy is denoted in positive months. Her disease remains well controlled on niraparib augmented by local therapies at 63 months.

Metastatic pulmonary disease became apparent on imaging later that year, and she received a course of doxorubicin monotherapy. Six months later, her progressive disease was treated with pazopanib with crossover to sapanisertib (MLN0128) on Alliance A091304 (NCT02601209), but she had disease progression after three months of each agent. She then took a break from systemic therapy and had serial targeted radiation to growing lesions in her lungs, vertebrae, rib, and brain, and CT-guided cryotherapy to perisplenic, anterior abdominal, and subcutaneous nodules. She resumed systemic therapy, but her disease continued to advance, with progressive disease after one cycle each of temozolomide, then trabectedin.

Cervical vertebral biopsy tissue was submitted for NGS (StrataNGS), which revealed *ERBB2* p.R896H mutation (51% variant allele frequency) as well as *BRCA2* deep deletion (exons 2-18), *RB1* deep deletion (exons 19-27) and *TP53* p.W91Sfs*56 mutation (76% variant allele frequency); subsequent FISH testing did not reveal amplification of *ERBB2* (HER2) in her tumor. Biomarker analysis showed microsatellite stability, low tumor mutational burden (1 mut/Mb), and low PD-L1 status (RNA expression score 6). She underwent left hip hemiarthroplasty following a pathologic fracture.

She was initiated on niraparib 300 mg twice daily in response to her homologous recombination repair deficiency (32) and the drug’s CNS penetration. She underwent palliative left femoral neck radiation (3D CRT, 20 Gy/5 fractions) within the first month of niraparib. Over the next six months, niraparib was reduced to 100 mg twice daily due to fatigue. She also received two courses of palliative cryoablation of four subcutaneous nodules. She experienced a niraparib treatment interruption for left hip arthroplasty repair following a displaced trochanteric fracture. She has been on niraparib for approximately 63 months with prolonged disease control and ongoing partial response.

Germline genetic testing has been recommended; the patient has not pursued it.

## 4 Discussion

These three cases illustrate several important points in the management of STS using precision medicine. First, we report two mutations not previously described in the literature, *GOPC-ROS1* fusion in angiosarcoma and *EML4-ALK* fusion in MPNST. *CEP85L/ROS1* was the first gene fusion described in angiosarcoma and the only *ROS* rearrangement detected in 34 angiosarcomas (33). No *ROS1* fusions were detected in 101 angiosarcomas (Supplemental material (34)). Two case reports describe CD34 and S100 positive spindle cell tumors with MPNST features that contain an *EML4-ALK* rearrangement (35, 36), but no fusions were detected in 50 MPNSTs (34). Much remains to be learned about the molecular profiles of these rare cancers, and sequencing can reveal unexpected and actionable mutations that could meaningfully improve patient outcomes.

While only about a third of STS tumors may have currently actionable mutations (16), the potential benefit for individual patients can be profound. With available palliative chemotherapies, the median overall survival for patients with metastatic non-cutaneous angiosarcoma, malignant peripheral nerve sheath tumor (MPNST), and uterine leiomyosarcoma is 3 months (12), 8 months (13), and 19.4 months (14), respectively. Our patients’ corresponding periods of disease control with targeted agents and local therapies of 12, 48, and 63 months underscore the necessity of continued pursuit of precision medicine solutions in this heterogeneous group of cancers.

Although our patients had partial responses, they were not uniformly durable. This suggests that suppression of a driver mutation clone by systemic therapy alone in sarcoma is insufficient for long-term disease control. Similarly, in ALK-rearranged NSCLC, benefits have been observed with local ablative therapy and continuation of ALK inhibitors post-progression in the setting of oligometastatic disease (41). Our cases illustrate that local therapies can be critical adjuvants in achieving good outcomes for aggressive sarcomas.

Another limitation of our NGS-based approach was the low yield (VAF 0.2%) of the *ROS1 G2032R* resistance mutation on liquid biopsy despite a high disease burden. Repeat tissue biopsy attempt was considered, but the clinical context, pretest probability, and mutation identification on non-invasive testing were deemed sufficient to accept the result. TRIDENT-1 results showed 59% of patients with a *ROS1 G2032R* mutation and 38% of patients with brain metastases with prior *ROS1* TKI use responded to repotrectinib (29). Many resistance mechanisms have not yet been elucidated. It is possible that a different mechanism was driving our patient’s disease resistance, which would explain her poor response to repotrectinib.

A third challenge in bringing novel therapies into sarcoma care is the lack of reliable biomarkers to assess responsiveness to immune checkpoint blockade (ICB). Our patient’s breast tumor may have had PD-L1 IHC staining performed through a triple-negative breast cancer testing cascade. PD-L1 IHC expression is not routinely performed in sarcomas, nor does it consistently correlate with immunotherapy responses in these cancers (24, 42, 43). Tissue submitted for NGS is limited to PD-L1 RNA expression, which is not clinically validated. In the years since our patient pursued ICB, preliminary results from Alliance A091902 showed 67% (6/9) response rate to cabozantinib and nivolumab in taxane-pretreated, noncutaneous angiosarcoma (44). Work is ongoing to validate alternative gene signatures to effectively predict response to ICB in sarcomas (45).

Current NCCN guidelines for STS still recommend weighing the utility and timing of NGS testing on an individual basis (31). Beneficial scenarios to consider include active consideration of clinical trials requiring sequencing, exhaustion of standard therapies, and specific histologies known to carry actionable mutations (31). Similarly, after showcasing the potential of NGS to refine sarcoma diagnoses, produce actionable results, and examples of durable disease control from NGS-informed targeted therapies, the authors agreed that this testing should be limited to select clinical cases (16). Reasons cited include cost and resource limitations. However, they acknowledged that the genomic landscape derived from heavily pre-treated, refractory disease specimens biases our understanding (16). The genomic diversity of these rare, diverse tumors continues to be uncovered, and an equitable mechanism for selective testing has not been established. Notably, advanced cancer patients with low socioeconomic status, Black race or Hispanic Ethnicity, and Medicaid or Medicare coverage experience a longer time to NGS testing (46).

Importantly, two of our three patients faced barriers to accessing clinical trials. This underscores the importance of access and availability of clinical trials in rare tumors. Efforts such as the national TAPUR trial, supported by ASCO and several other partners, may offer a path forward, but our experience indicates that slot availability remains a barrier to enrollment. Many of the challenges posed by the COVID-19 pandemic further impeded trial access, a problem that is improving at our institution and at many others. Notably, a GOG/Alliance trial (NCT05432791) completed accrual to evaluate a PARP inhibitor in combination with temozolomide in advanced uterine leiomyosarcoma, providing an opportunity to evaluate the findings from NCI Protocol 10250 (37) in a randomized study.

Our cases demonstrate that the administrative burden of accessing targeted therapies outside clinical trials can lead to clinically detrimental treatment delays. Real-world data show that delays in targeted therapy initiation for advanced NSCLC are associated with an increased risk of death (38, 39). A national online survey showed younger patients with employer insurance plans and those with advanced disease are most likely to experience cancer treatment delays due to prior-authorization negotiations; targeted therapy approval was most likely to require direct patient involvement (40).

We agree with the careful consideration of the potential benefits of NGS in sarcoma care. At our institution, we are moving toward universal, comprehensive genomic profiling for patients with sarcoma. Others have called for and begun to use this approach (47, 48). In localized disease, we hope this will provide improved diagnostic utility. Additionally, in high-risk disease, it enables proactive discussions of potential next lines of therapy and can expedite initiation of appropriate therapy upon disease progression. In metastatic disease, as illustrated above and echoed in an ASCO provisional clinical opinion (49), this may provide real, tangible benefits for patients afflicted by these challenging diseases. More frequent testing at the time of diagnosis could enable identification of biomarkers of response to standard cytotoxic chemotherapy, avoiding toxicity without oncolytic benefit. Further efforts to characterize sarcoma biology and expand access to clinical trials for rare tumors are needed to advance the field, ideally returning it to its origins at the cutting edge of precision medicine.

## Data Availability

The original contributions presented in the study are included in the article/supplementary material. Further inquiries can be directed to the corresponding author/s.
